# Group‐guided individual functional parcellation of the hippocampus and application to normal aging

**DOI:** 10.1002/hbm.25662

**Published:** 2021-09-16

**Authors:** Jiang Zhang, Dundi Xu, Hongjie Cui, Tianyu Zhao, Congying Chu, Jiaojian Wang

**Affiliations:** ^1^ College of Electrical Engineering Sichuan University Chengdu China; ^2^ National Key Laboratory of Cognitive Neuroscience and Learning Beijing Normal University Beijing China; ^3^ State Key Laboratory of Primate Biomedical Research, Institute of Primate Translational Medicine Kunming University of Science and Technology Kunming China

**Keywords:** affinity propagation, aging, hippocampus, individual parcellation, resting‐state fMRI

## Abstract

Aging is closely associated with cognitive decline affecting attention, memory and executive functions. The hippocampus is the core brain area for human memory, learning, and cognition processing. To delineate the individual functional patterns of hippocampus is pivotal to reveal the neural basis of aging. In this study, we developed a group‐guided individual parcellation approach based on semisupervised affinity propagation clustering using the resting‐state functional magnetic resonance imaging to identify individual functional subregions of hippocampus and to identify the functional patterns of each subregion during aging. A three‐way group parcellation was yielded and was taken as prior information to guide individual parcellation of hippocampus into head, body, and tail in each subject. The superiority of individual parcellation of hippocampus is validated by higher intraregional functional similarities by compared to group‐level parcellation results. The individual variations of hippocampus were associated with coactivation patterns of three typical functions of hippocampus. Moreover, the individual functional connectivities of hippocampus subregions with predefined target regions could better predict age than group‐level functional connectivities. Our study provides a novel framework for individual brain functional parcellations, which may facilitate the future individual researches for brain cognitions and brain disorders and directing accurate neuromodulation.

## Graphical abstract

A new individual parcellation approach was developed. Individual variations were associated with coactivation patterns of cognitive functions. The individual functional connections can better predict age.

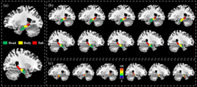

## 1 INTRODUCTION

Aging not only affects individuals' lifestyle, but also gradually modulates brain structures and functions, especially attention and memory capacities (Damoiseaux, 2017; Onoda, Ishihara, & Yamaguchi, 2012; Mather & Carstensen, 2005; Mcdonough, Wood, & Miller, 2019). The aberrant aging trajectory is related to the onset of Alzheimer's disease, a syndrome with severe cognitive decline (Agosta et al., 2012; Cha, Hang, Kim, Sang, & Lee, 2013; Conwell et al., 2018; Wu et al., 2016).

The hippocampus is pivot for cognition, learning, and memory processing in the human brain (Bartsch & Wulff, 2015; Duzel, Praag, & Sendtner, 2016; Knierim, 2015; Max, 2017). During aging, declined overall volume of hippocampus, especially in people after 60 years has been widely reported (Fjell, Mcevoy, Holland, Dale, & Walhovd, 2014; Flores, Joie, & Chételat, 2015). The previous reports showed that elevated age‐related coupling between the bilateral hippocampus is associated with lower episodic memory performance indicating hippocampus collapse leads to memory loss and potentially causes a fast transition to AD in elderly individuals (O'Brien et al., 2010; Salami, Pudas, & Nyberg, 2014). At the subregion‐level, recent studies found lower functional connectivity between posterior hippocampus and medial prefrontal cortex in venerable age compared to younger age population (Damoiseaux, Viviano, Yuan, & Raz, 2016; Wang et al., 2010). All these studies demonstrated that to delineate the functional patterns of hippocampus is important to reveal the neural basis of cognition and memory decline during aging (Andrewshanna et al., 2007; Koch et al., 2010).

Hippocampus is a functionally heterogamous area with different functional subregions having different functions (Das et al., 2011; Zarei et al., 2013; Zhong et al., 2019). The anterior hippocampus is mainly involved in emotion processing while the posterior hippocampus primarily participates in spatial memory (Blum, Habeck, Steffener, Razlighi, & Stern, 2014). Thus, to uncover the aging effects at the subregion‐level may better identify the neural mechanism of aging. Although subregions of the hippocampus have been defined with cytoarchitecture or connectivity‐based parcellation approaches, however, different parcellation studies proposed different parcellation schemes for hippocampus (Amunts et al., 2005; Cheng & Fan, 2014; Cheng, Zhu, Zheng, Liu, & He, 2020; Robinson et al., 2015; Zhong et al., 2019). Moreover, all the previous parcellation schemes for hippocampus are based on group‐level mapping and thus are hardly to reflect the individual differences.

Individual differences in cognition and behaviors have been widely reported in neuroscience researches (Brown, 2017; Tavor et al., 2016). The brain structural and functional variability across individuals has also been identified in recent literatures (Mueller et al., 2013). The individual brain functional parcellation and functional networks mapping were developed and employed to reveal the functional underpinnings for brain cognitions and disorders (Han et al., 2019; Wang, Buckner, et al., 2015). However, all the existing individual brain mapping approaches are based on the predefined prior brain atlas to guide parcellation, and thus they are constrained by the parcellation schemes of the used atlases (Han et al., 2019; Li et al., 2019; Wang et al., 2018). Although consistent anatomical and functional topological architecture of some brain areas have been documented, the topological variations between structural, anatomical, and functional parcellations have been demonstrated (Caspers et al., 2008; Ruschel et al., 2014; Wang et al., 2012; Wang et al., 2017). Thus, atlases‐free individual parcellation and functional mapping are essential and may better characterize the individual functional organization to delineate the individual differences compared to group‐level mapping.

In this study, we proposed a new individual brain parcellation approach using affinity propagation clustering which is able to automatically define the number of clusters (Frey Brendan & Detbert, 2007). Then, we validated the superiority of our proposed method by measuring the functional similarities compared to group‐based parcellation results. In addition, the relationship between individual variations and coactivation patterns of hippocampus mostly related cognitive functions were evaluated. Finally, we applied this approach to define individual functional subregions of hippocampus in aging population and to test whether individual parcellation could better predict age than group‐level parcellation.

## 2 MATERIALS AND METHODS

### 2.1 Subjects

A public adult lifespan resting‐state functional magnetic resonance imaging (rs‐fMRI) data (Southwest University Adult lifespan Dataset, SALD) was accessed through f1000 project (http://fcon_1000.projects.nitrc.org/indi/retro/sald.html). By excluding the bad fMRI data with large head motion (see the following fMRI preprocessing), a total of 262 healthy subjects (101 males/161 females, age range of 19–75 years, mean and *SD* = 42.03 ± 16.73 years) with high quality rs‐fMRI were finally used in this study.

### 2.2 Resting‐state fMRI data acquisition

The rs‐fMRI data were scanned using a Siemens 3T Tim Trio MRI scanner with echo planar imaging sequence. The subjects were instructed to lie down, close their eyes, and rest without thinking anything and not fall asleep. The acquisition parameters were the followings: TR = 2,000 ms, TE = 30 ms, voxel size = 3.4 × 3.4 × 3 mm^3^ with 1 mm gap, 32 axial slices, and 242 volumes. The detailed information about subjects and rs‐fMRI scanning parameters can be found in a previous study (Wei et al., 2018).

### 2.3 Resting‐state fMRI data pre‐processing

The preprocessing of resting‐state fMRI data including the following steps: discarding the first 10 volumes to facilitate magnetization equilibrium; realigning all the remained volumes to the first volume to correct head motion; normalizing to the standard EPI template in MNI space; regressing out Friston 24‐parameter model of head motion, white matter, cerebrospinal fluid and global mean signals; filtering with a temporal band‐pass of 0.01–0.1 Hz. To exclude the head motion effects, the data were discarded if the head‐movement exceeded 2 mm or 2° in any direction. Moreover, scrubbing was further used to eliminate the bad images (before two time points and after one time point) exceeding the pre‐set criteria (frame displacement [FD], FD < 0.5) for excessive motion. If the deleted number of volumes exceeding half of the time points, that is, 116 volumes for each subject, this subject was discarded for the following analysis. Under these criteria, 232 subjects were excluded and the remained 262 subjects were used for analyses.

### 2.4 Hippocampus definition

To define the individual functional subregions of hippocampus, the human bilateral hippocampus seed masks were defined using Harvard‐Oxford cortical atlas with 25% probability (Desikan et al., 2006). After obtaining the hippocampus masks, the bilateral masks were downsampled into 3 mm cubic voxel to calculate functional connectivity for parcellation.

### 2.5 Group‐guided individual parcellation of hippocampus

To achieve individual parcellation of hippocampus, we first performed the group‐level parcellation of hippocampus across all the subjects. For group‐level parcellation, we first calculated the whole brain functional connectivity map for each voxel within hippocampus. Next, the similarity for the functional connectivity maps of every pair of voxels within the hippocampus was defined using eta^2^ (Wang, Yang, et al., 2015; Wang et al., 2016), and a similarity matrix *S* for each subject was obtained. Then, all the similarity matrixes were averaged to obtain one similar matrix which was clustered to obtain the parcellation results of hippocampus. To automatically identify the optimal number of clustering, the affinity propagation method (Frey & Dueck, 2007) was employed to segment the similarity matrix.

The AP algorithm requires two input parameters: input similarity matrix *S*, in which *S(i*,*k)* is the negative value of the squared Euclidean distance between points *i* and point *k* in the similarity matrix (for similarity matrix *X* = {*x*_1_,*x*_2_,…,*x*_*N*_}, $s\left(i,k\right)=-{\left\|x_{i}-x_{k}\right\|}^{2}$, *i* ≠ *k*, *i*, *k*∈{1,2,…,*N*}); and preference “P,” recommended as the median of *S(i*,*k)* when there is no prior, determines the number of clustering (Frey & Dueck, 2007). The algorithm takes all data points as potential exemplars and calculates the following two messages including the “availability” *a(i*,*k)* and the “responsibility” *r(i*,*k)*) to characterize the appropriateness of exemplars selection for the data points iteratively to obtain the optimal clustering results (Zhang, Li, et al., 2011; Zhang, Tuo, et al., 2011). The number of clusters was finally determined when the iterative process converges. The details of AP clustering are as follows (Xia et al., 2008):

Step 1: initialize the availabilities:

$$a\left(i,k\right)=0.$$

Step 2: update the responsibilities:

$$r\left(i,k\right)\leftarrow S\left(i,k\right)-\max_{k's.t.k'\neq k}\left\{a\left(i,k'\right)+S\left(i,k'\right)\right\}$$

Step 3: update the availability:

$$a\left(i,k\right)\leftarrow \min\left\{0,r\left(k,k\right)+\sum_{i's.t.i'\neq i,k}\max\left\{0,r\left(i',k\right)\right\}\right\}$$

$$a\left(k,k\right)\leftarrow \sum_{i's.t..i'\neq k}\max\left\{0,r\left(i',k\right)\right\}$$

Step 4: iterations convergence terminate:

$$c_{i}\leftarrow \underset{k}{\arg\max}\left\{r\left(i,k\right)+a\left(i,k\right)\right\}$$

For parameter of preference “P,” the recommended value usually does not obtain a satisfactory result. In our study, we used a semisupervised algorithm for step optimization to search the optimal “P.” The optimal “P” value was determined by identifying the largest silhouette value across different “P” values (Kaufman & Rousseeuw, 1990). At a specific clustering solution, silhouette value, *sil*, is defined as follows:

$$\mathrm{sil}\left(i\right)=\frac{b\left(i\right)-a\left(i\right)}{\max\left\{a\left(i\right),b\left(i\right)\right\}}$$

where *a* represents intracluster similarity, that is, the average distance between data point *i* and the other points in a specific cluster, and *b* is the interclusters similarity, that is, average distance between data point *i* in one cluster and all the data points in the other cluster. The average of the silhouette values is defined:

$$\mathrm{AS}=\frac{1}{N}\sum_{i=1}^{N}\mathrm{sil}\left(i\right)$$

where *N* is the total number of data points. “AS” reflects the quality of clustering results varying from 0 to 1, and a larger “AS” represents better clustering quality. To initialize the “P” value, *S*_med_ and *S*_min_ as median and min of *S*(*i*,*k*) were calculated, respectively. The “P” changed from *S*_med_ recommended by the original paper to the values of the end of search (*S*_med_ + step × number of steps, where step = *S*_min_/*T*, *T* is a random value to determine the length of step, *T* = 100 used in this study). If the “AS” values go smooth from one step to all the other steps behind it, the corresponding “P” value is considered to be optimal. The smooth of the changing “AS” values was characterized by the gradient values calculated as the latter value minus the previous value, and no blunt change of the gradient value was considered to be smooth. The number of clusters corresponding to the optimal “P” was taken as the optimal group‐level parcellation results.

Next, the group‐level parcellation of hippocampus was used to guide the individual parcellation using Litekemans method which has fast speed and high accuracy (http://www.cad.zju.edu.cn/home/dengcai/Data/code/litekmeans.m). The mean whole brain functional connectivity map of each subregion of hippocampus yielded by group‐level parcellation was first calculated and taken as initial clustering centers across all the voxels within each subregion and across all the subjects. Finally, the whole brain functional connectivity maps of all the voxel in hippocampus in each subject were clustered to achieve individual parcellation of hippocampus.

### 2.6 Validation of the individual parcellation approach

To test and validate the reliability of the developed group‐guided individual functional parcellation approach, we applied this method to parcellate supplementary motor area (SMA), a brain region that has been widely used to test the applicability and reliability of resting‐state fMRI‐based functional parcellation because of its established functional architecture (Johansen‐Berg et al., 2004; Kim et al., 2010; Zhang et al., 2015). SMA mask was defined using automated anatomical labeling template (Tzourio‐Mazoyer et al., 2002). Then, individual parcellation of SMA was performed using our developed group‐guided individual parcellation approach. Both two‐ and three‐way parcellations of SMA were performed in both group and individual levels referring to previous studies.

### 2.7 Functional similarity validation

To validate the superiority of individual parcellation results compared to group‐level parcellation, intraregional functional connectivities and time series similarities were calculated in this study. The intraregional functional connectivity similarity is the average Pearson correlation coefficients between any pair of voxels in each subregion. The intraregional time series similarity is characterized using Kendall's coefficient concordance (KCC) of all the voxels' time series in a specific subregion (Kendall, 1990).

$$\mathrm{KCC}=\frac{\sum {\left(R_{i}\right)}^{2}-n{\left(\bar{R}\right)}^{2}}{\frac{1}{12}K^{2}\left(n^{3}-n\right)}$$

where $R_{i}$ is the sum rank of the *i*th voxel in a specific subregion of hippocampus, $\bar{R}=\left(\left(n+1\right)K\right)/2$ is the mean of the $R_{i}$, *K* is the number of voxels in a specific subregion, and *n* is the number of total time points.

### 2.8 Relationship between individual parcellation variations and coactivation patterns

To explore relationship between the variations of individual parcellation and the coactivation patterns of hippocampus most related cognitive functions including cognitive memory, episodic recall, and emotion of angry. Meta‐analyses of cognitive memory, episodic recall, and emotion of angry were performed in the BrainMap database. To obtain the peak ALE value of the hippocampus associated with cognition memory, episodic recall, and angry emotion, the meta‐analysis was employed in the BrainMap database. The search criteria as follows: the behavior domain was cognition memory and emotion of angry; the paradigm class was episodic recall; the imaging modality was fMRI; and the experiment content was normal mapping. Meta‐analysis of cognition memory identified 386 papers, 1,233 experiments. Meta‐analysis of episodic recall identified 22 papers, 97 experiments, and meta‐analysis of emotion of angry identified 37 papers, 97 experiments. Next, activation likelihood estimation (ALE) was employed to map the coactivation patterns for the three functions. Then, ALE value of each voxel in hippocampus was extracted and voxel‐wise correlation analyses were performed between ALE values and individual variations.

### 2.9 Age prediction

To further validate the superiority of individual functional parcellation, the individual and group‐level functional connectivities were separately taken as features to predict individual ages. To define hippocampus involved functional networks, the voxel‐wise whole brain functional connectivity analysis of bilateral hippocampus was first performed to define the target brain areas. One‐sample *t* test was used to identify the significantly connected brain areas with hippocampus. The significant level was corrected using family discovery rate method with *p* < .001, and minimum cluster size > = 100.

Next, the functional connections of each hippocampus subregions yielded by individual and group‐level parcellation with these target brain areas were calculated and taken as features for prediction. Relevance vector regression method which showed better performances than other prediction methods was applied to predict the age (Cui & Gong, 2018; Cui et al., 2016). A 10‐fold cross‐validation strategy was used to estimate the generalization ability of the prediction mode (Tang et al., 2018). Pearson correlation coefficient between the real age and predicted age was calculated to depict the prediction performance. The mean absolute error (MAE) values between real and predicted age were also calculated to evaluate prediction.

### 2.10 Age‐related functional connections with hippocampus

To explore age associated functional connections with hippocampus, correlation analyses were performed between seed‐to‐target functional connections and age. The seed to targets functional connections measured with Pearson's correlation coefficients between time series were calculated for individual and group‐level hippocampus subregions. The Fisher *r*‐to‐*z* transformation was used to change the *r* value to *z* value. Finally, correlation analysis between each individual and group‐level functional connectivity and age was performed, and the significant level was set at *p* < .05 with Bonferroni correction.

### 2.11 Comparisons with iteration adjusted individual parcellation approach

We also compared our method with the previous iteration adjusted individual parcellation approach developed by Wang, Buckner, et al. (2015). First, a group‐level parcellation of hippocampus was obtained and used to guide individual parcellation. Second, individual parcellation of hippocampus for each subject was executed using iteration adjusted individual parcellation approach proposed by Wang, Buckner, et al. (2015). To compare the performances of the two individual parcellation approaches, voxel‐wise overlap degree of each hippocampus subregion, correlations between voxel‐wise individual variations and ALE values of the coactivation patterns for cognitive memory, emotion of angry, and episodic recall, and age prediction with individual functional connectivities of hippocampus subregions were investigated.

## 3 RESULTS

### 3.1 Individual functional parcellation of the hippocampus

**FIGURE 1:**
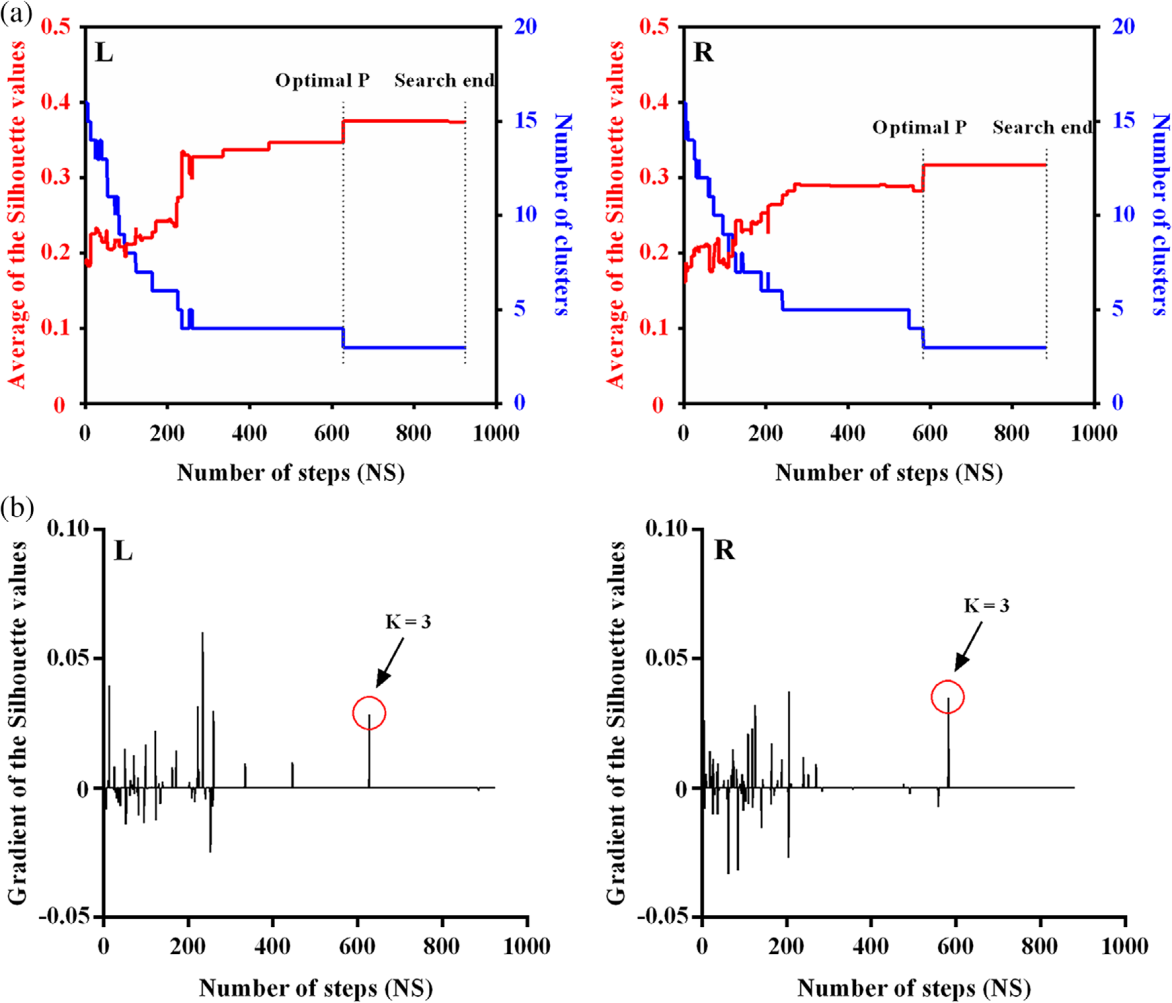
The criteria to determine the optima number of clusters. The average silhouette values and the changes of gradient were used to select the number of clusters. (a) The average silhouette values identified the optimal three‐way parcellation for bilateral hippocampus which show stable changes of the maximum average silhouette across more than 300 steps. (b) The gradient value of average silhouette values across all the steps were depicted and no blunt change of gradient was found when hippocampus were parcellated into three subregions (*K* = 3). Thus, the optimal three‐way parcellation of hippocampus was finally used in this study

**FIGURE 2:**
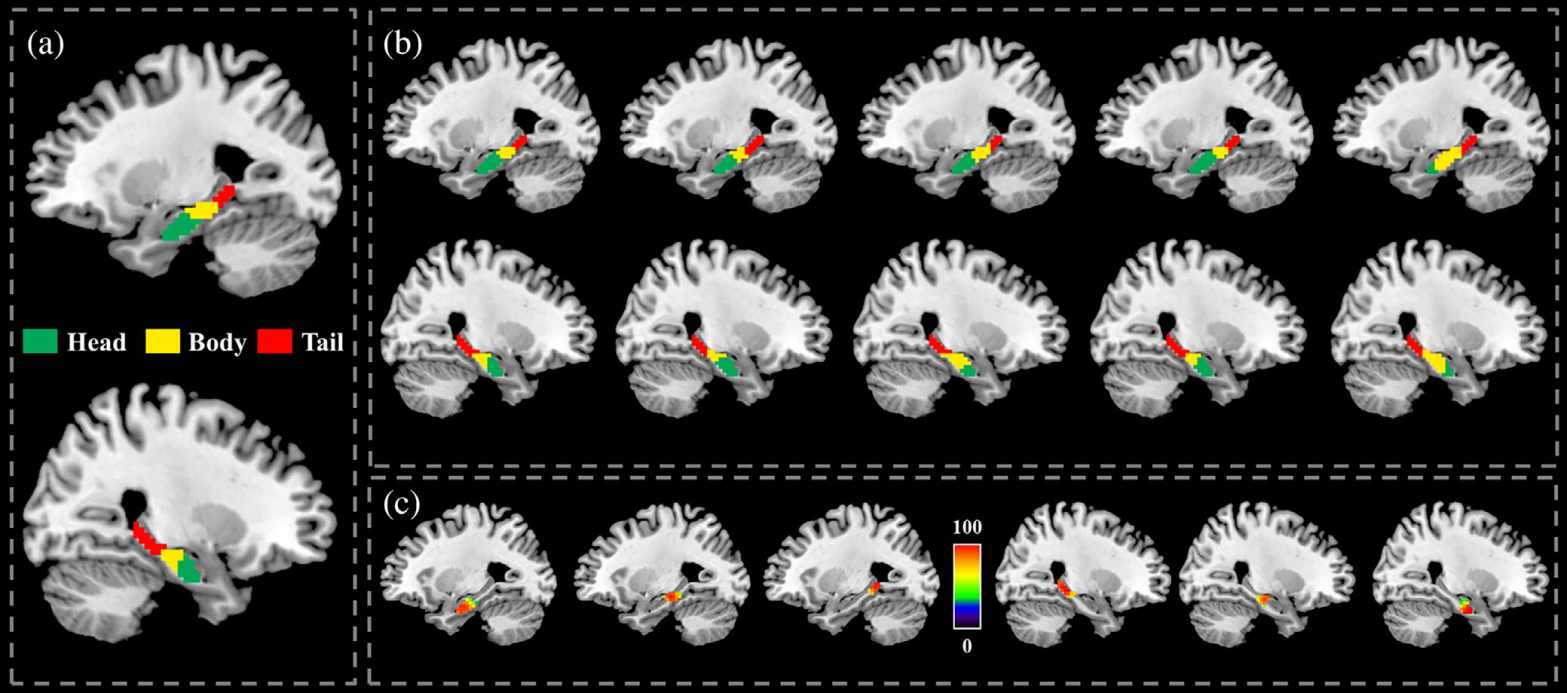
Group‐level and individual hippocampus parcellation results. (a) Three‐way group‐level parcellation of bilateral hippocampus identified head, body, and tail subregions from anterior to posterior direction. (b) Five randomly selected individual parcellation of hippocampus were shown. The individual parcellation of hippocampus showed obvious variability of size and location of each subregion between individuals. (c) Voxel‐level overlap degree of each hippocampus subregion across all the subjects was calculated. Although individual variations, the high overlap degree for each subregion was observed except the intersected part between subregions

To determine the number of clusters, the maximum average silhouette value and smooth variation of gradient of the average silhouette were taken as the criteria to terminate searching the optimal preference parameter (Figure 1a,b). Based on the criteria, the optimal three‐way group‐level parcellation of bilateral hippocampus was found. The three‐way group‐level parcellation identified head, body, and tail of hippocampus (Figure 2a). Using the group‐level parcellation results of hippocampus as prior information, the individual parcellation results for hippocampus were obtained, and five randomly selected individual parcellation results are shown in Figure 2b. The obvious individual variations of hippocampus parcellation results could be observed.

To quantitatively describe the individual variations, we calculated the overlap degree at the voxel level for each subregion across all the subjects (Figure 2c). The tail part in bilateral hippocampus show high overlap degree, while the overlap degree in body and head part of hippocampus is relatively lower than tail part, especially at the intersected part.

To validate our developed group‐guided individual parcellation approach, we adopted the same procedures to parcellate SMA in which the functional topography has been well established. The group‐guided individual functional parcellation identified similar group‐level functional topography of this areas identified by previous studies (Crippa et al., 2011; Johansen‐Berg et al., 2004; Kim et al., 2009; Zhang et al., 2015). The individual variations of functional subregions in SMA could be obviously observed in five randomly selected subjects (Figure S1).

### 3.2 Validation of functional similarity

**FIGURE 3:**
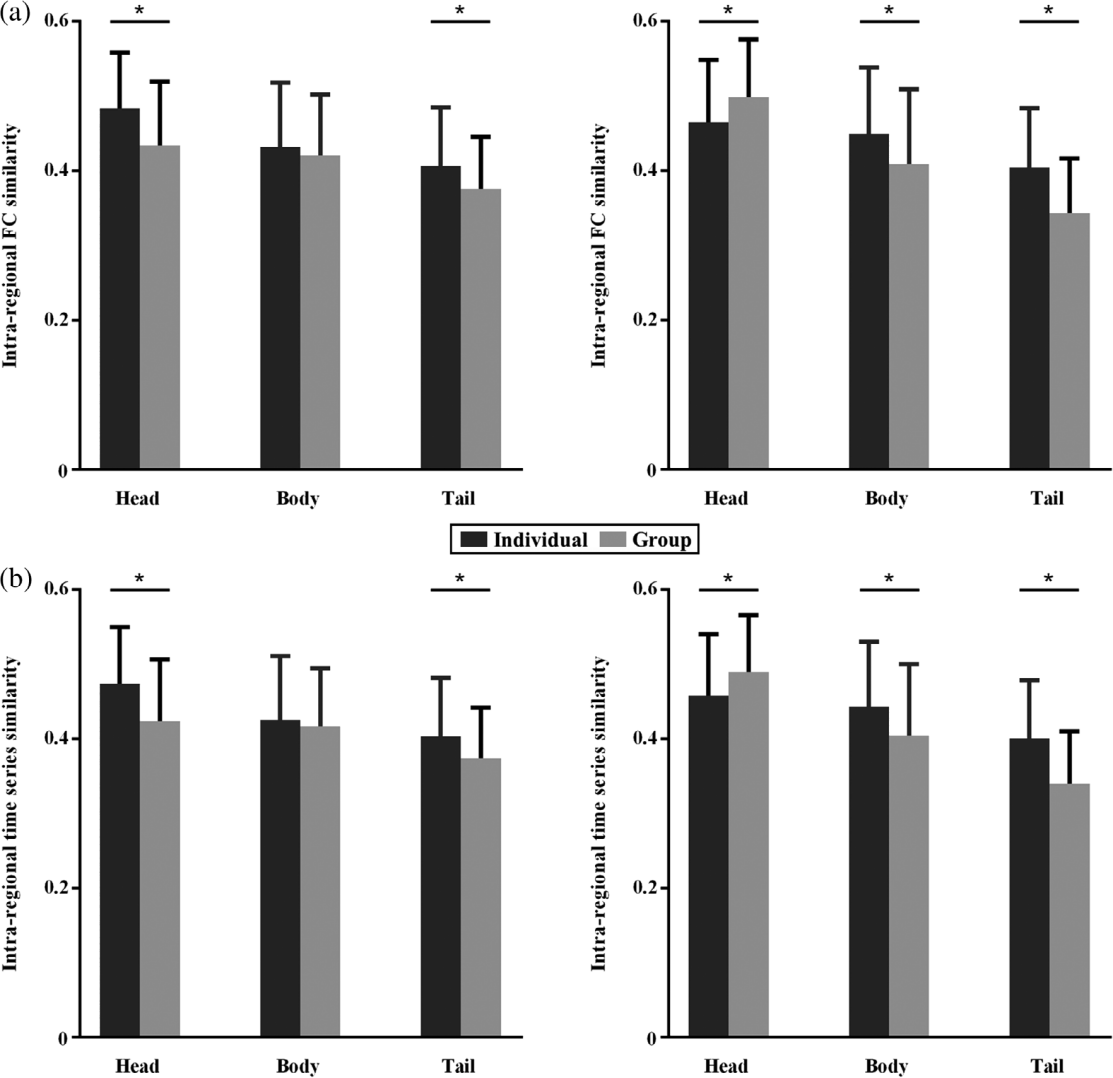
Functional similarities comparison between the group and individual parcellation results. The intraregional functional connectivity (a) and time series (b) similarities between any pair of voxels within each hippocampus subregion were calculated. Almost all the functional connectivity and time series similarities of hippocampus subregions at the individual level are higher than that at group level except the head part of right hippocampus in which the intraregional functional connectivity and time series similarities at group level are higher than individuals. (*represents *p* < .05 after Bonferroni corrected)

Intraregional functional connectivity and time series similarities analyses identified significantly higher similarities in individual than group‐level parcellation results of hippocampus subregions except for the right head part of hippocampus which showed higher functional connectivity (Figure 3a) and time series similarities (Figure 3b) in group level than individual parcellation results. The functional connectivity and time series similarities results indicated that individual parcellation approach could better identify functionally homogenous subregions compared to group‐level parcellation method.

### 3.3 Individual parcellation variations associated with coactivation patterns

**FIGURE 4:**
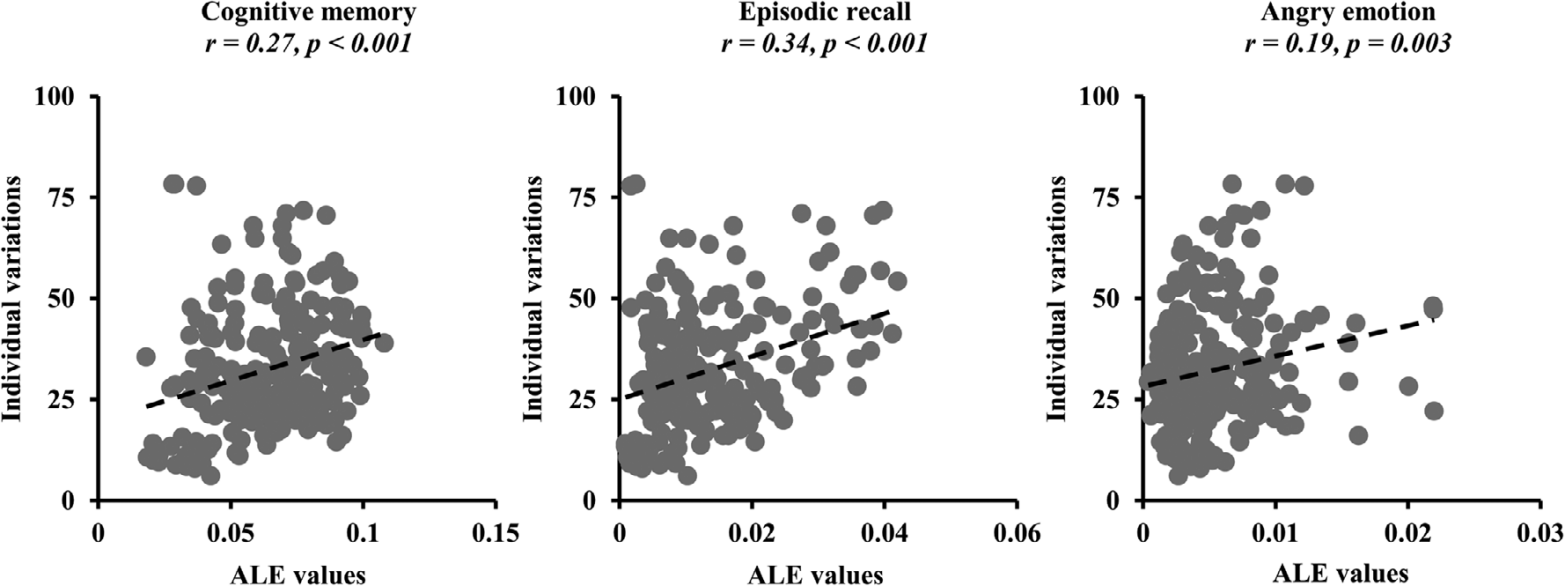
Correlations between individual variations and activities patterns of cognitive functions within hippocampus. Voxel‐wise correlation analyses between individual variations of hippocampus parcellation results defined using our proposed approach and coactivation patterns, that is, activation likelihood estimation (ALE) values, of cognitive memory, episodic recall, and emotion of angry of hippocampus were performed. Significant correlations between individual variations and ALE values of cognitive memory, episodic recall, and emotion of angry were found

Voxel‐wise correlation analyses identified significant associations of individual variations with ALE values of cognitive memory (*r* = .27, *p* < .001); episodic recall (*r* = .34, *p* < .001); and emotion of angry (*r* = .19, *p* = .003) (Figure 4). These results indicated that the individual variations of hippocampus parcellations were closely associated with activity patterns of cognitive functions of hippocampus.

### 3.4 Age prediction

Based on the whole brain functional connectivity mapping of hippocampus, eight target brain areas showing significantly functional connections with hippocampus were defined (Figure S4 and Table S1). After obtain the eight target brain areas, the functional connectivity between each individual and group‐level hippocampus subregion and each target was calculated.

**FIGURE 5:**
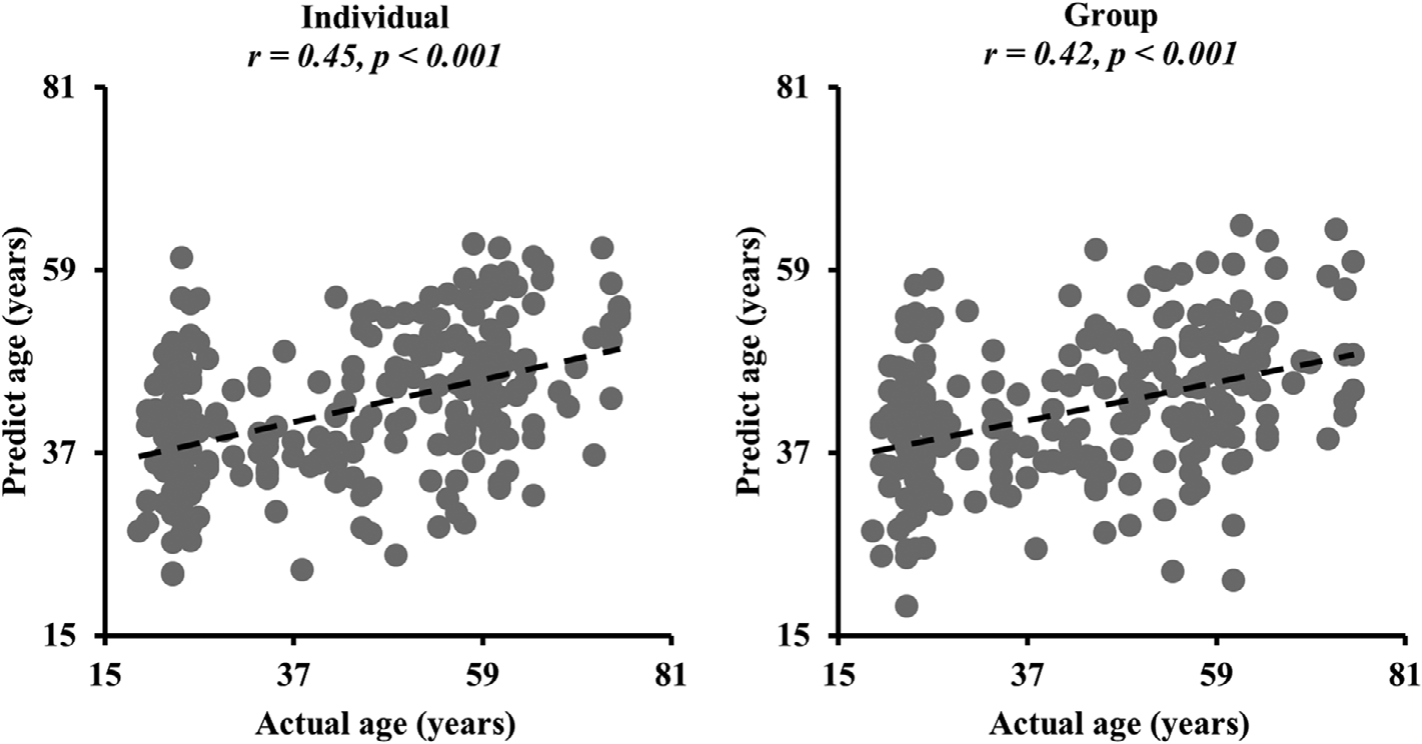
Age prediction using individual and group‐level functional connections. Both individual and group‐level functional connectivities were separately taken as features to predict age. The Pearson correlation coefficient was used to characterize the prediction performance. The individual functional connections showed better performance, that is, higher correlation coefficient, than group‐level functional connections‐based prediction

To explore whether individual functional connectivities can better predict age than group‐level functional connections of hippocampus subregions, the individual and group‐level functional connections were separately used as features to predict ages. Compared with group‐level functional connections‐based prediction results (*r* = .42, *p* < .001), individual functional connections (*r* = .45, *p* < .001) exhibited better performance (Figure 5). The MAE values is smaller using individual functional connections‐based prediction compared with group‐level‐based prediction (individual MAE = 12.5, group MAE = 12.95).

### 3.5 Associations between hippocampus connectivities and age

Correlation analyses showed that the functional connection between the body part of left hippocampus and left retrosplenial cortex showed significant correlation with age only at the individual‐level (Figure S6a). The functional connectivities of right tail part of hippocampus with left cerebellum and left retrosplenial cortex, and functional connections of head part of left hippocampus with right medial prefrontal cortex were significantly correlated with ages only at group level (Figure S6b). Moreover, significant correlations between age and the functional connections of left body part of hippocampus with left cerebellum and left superior frontal gyrus, and the functional connections of head part of right hippocampus with right medial prefrontal cortex were found at both individual and group levels (Figure S6c).

### 3.6 Comparison with iteration adjusted individual parcellation method

To compare the performance of our and Wang, Buckner, et al. (2015) developed individual parcellation methods, voxel‐level overlap degree of individual hippocampus subregions were calculated using both methods. Iteration adjusted individual parcellation (Figure S2b) showed high overlap degree across the subjects compared with our individual parcellation approach (Figure S2a,c). The result suggested that our proposed method can better delineate individual variability.

In addition, Pearson correlation coefficient of voxel‐wise individual variations defined with iteration adjusted individual parcellation approach and ALE values of cognitive memory, episodic recall, and emotion of angry were calculated. Only the coactivation patterns of cognitive memory and episodic recall showed correlations with individual variations of hippocampus, the coactivation pattern of emotion of angry did not exhibited significant associations with individual variations (Figure S3). Importantly, the correlation coefficients of individual variations defined using iteration adjusted individual parcellation approach are lower than our proposed individual parcellation approach.

Finally, individual functional connections of individual hippocampus subregions defined using iteration adjusted individual parcellation approach were taken as features to predict age. Correlation analysis showed that individual functional connections can well predict age but with lower correlation coefficient compared to our method (Figure S5).

## 4 DISCUSSION

This study proposed a novel individual brain parcellation approach based on the semisupervised affinity propagation clustering using resting state fMRI. The proposed method was subsequently applied to parcellate hippocampus to study the aging effects on hippocampus. The hippocampus was successfully subdivided into three parcels in each individual guided by a three‐way group parcellation results. Functional connectivity and time signals similarity analyses demonstrated the superiority of individual parcellation compared to group results. In addition, individual variations were found to be positively correlated with coactivation patterns of hippocampus related cognitive functions. Finally, individual and group‐level functional connections of hippocampus subregions with predefined targets were employed to predict the ages and individual functional connections showing better performance than group‐level functional connections. Taken together, our study presented a novel individual functional parcellation approach, which may facilitate the future study to better investigate the individual cognitive functions and behaviors and guide individual accurate neuromodulation.

### 4.1 Affinity propagation algorithm for brain parcellation

Affinity propagation algorithm could automatically determine the number of clusters by setting the preference “*P*” value which is recommended as the minimum or median value of the input similarity matrix to obtain small or moderate number of clusters in the original affinity propagation method (Frey Brendan & Detbert, 2007). However, given that the recommended input parameter “*P*” usually does not work to achieve the ideal clustering results, especially for brain parcellation, thus, in this study, we set a range of “*P*” values and the optimal “*P*” value was selected with high silhouette values and small or smooth gradient variations across these silhouette values. Our findings demonstrated that the method to select the optimal number of clusters could obtain reliable parcellation results by comparing our findings with previous hippocampus parcellation results.

### 4.2 Individual brain parcellation

The human brain is a complex system with huge individual differences in brain structure and functions (Mueller et al., 2013). To map the individual functional atlas is prevalent to reveal the individual differences in behaviors and cognitions (Goulas et al., 2012; Nebel et al., 2014). With predefined 18 cortical networks as prior information, Wang, Buckner, et al. (2015) developed an individual cortical parcellation approach and demonstrated that the functional networks mapped by this approach were highly reproducible within subjects and effectively captured the variability across subjects. Subsequently, Wang et al. (2018) demonstrated that the individual cortical functional network connections mapped with individual cortical areas could well predict the positive, negative, and manic symptoms in schizophrenia and bipolar disorders. In addition, Li et al. using individual functional mapping approach found that the individual functional connections could better predict fluid intelligence than group‐level connections (Li et al., 2019). Recently, Meizhen et al. (2020) employed diffusion MRI and the Brainnetome atlas as prior information for individual anatomical connectivity‐based brain parcellation. However, all these studies to map individual functional subregions relied on the previous atlas as prior information, which may thus miss the important functional subregions not present in the used atlas and result in different brain atlas yielding different individual parcellation results. Additionally, iteration adjusted approach for individual parcellation developed by Wang et al. achieves individual mapping by measuring the functional similarity and this process stops when the similarity is below a predefined threshold. However, how to determine the similarity threshold is lack of gold standard and different thresholds may affect the final solutions. To avoid these problems, we developed a fully data‐driven approach without prior information as guide to map the individual functional atlas. Our method was applied to hippocampus and obtained reliable group and individual parcellation results.

### 4.3 Individual parcellation of hippocampus

The hippocampus is a critical brain region participating in human memory, cognition, and emotional processes (Bartsch & Wulff, 2015; Duzel et al., 2016; Knierim, 2015; Max, 2017). The structural and functional diversities suggest that the hippocampus can be divided into distinct functional subregions. Adnan et al. used k‐means method to parcellate the bilateral hippocampus into the anterior and posterior subregions based on different anatomical connectivity patterns (Adnan et al., 2016). The anterior hippocampus mainly connects to the bilateral amygdala, right temporal pole, and right orbitofrontal cortex while the posterior hippocampus primarily connects with the left dorsal posterior cingulate cortex, retrosplenial cortex, and right superior parietal lobule. Using resting‐state fMRI, hippocampus was also parcellated into head, body, and tail parts (Cheng et al., 2020; Zhong et al., 2019). Ge et al. (2019) adopted the covariance of gray matter volume to parcellate the bilateral hippocampus into seven morphologically different subregions based on high‐resolution structural image data. The corresponding subregions on both hemispheres exhibit similarities in function and structure, and the structural covariance pattern corresponds to the functional connectivity pattern. Using coactivation‐based parcellation approach, Robinson et al. parcellated the left hippocampus into three clusters and the right hippocampus into five clusters (Robinson et al., 2016). Based on combined cytoarchitecture and chemoarchitecture properties, the hippocampus was also parcellated into different subregions (Ding & Hoesen, 2015). Although the hippocampus was parcellated with different techniques, almost all of these parcellation results are at group level not considering the individual variations. In our study, we developed a group‐level guided individual parcellation of hippocampus, which could not only capture the population information but also characterize the individual variations.

### 4.4 Hippocampus and aging

The human brain undergoes a series of complex structural and functional decline during aging (Damoiseaux, 2017). Existing researches demonstrate that the overall gray matter volume of hippocampus in the elderly is significantly smaller than in healthy adults (Apostolova et al., 2012; Fleischman et al., 2014; Golomb et al., 1993; Miller & O'Callaghan, 2005), and the anterior hippocampus shrinks faster than the posterior hippocampus during aging (Chen et al., 2010).

Aging is also closely related to functional integrity of hippocampus, but the detailed relationship is still controversial (Blum et al., 2014). The functional connectivities between hippocampus and the default mode network including posterior cingulate cortex, medial prefrontal cortex, and lateral parietal cortex are negatively correlated with age. The functional connectivity between the left and right posterior hippocampus decreases with age (Damoiseaux et al., 2016). However, in another study, the coupling between the bilateral hippocampus was found to increase with age (Salami et al., 2014). In addition, the precuneus and bilateral medial temporal lobes were found to have stronger connections to the anterior hippocampus in young people, but have stronger connections to the posterior hippocampus in the elder (Blum et al., 2014). In spite of inconsistency of these findings, all the evidence suggests an important role of hippocampus in aging. In our study, we found the functional connectivities between hippocampus and medial prefrontal cortex, superior frontal gyrus, retrosplenial cortex, and cerebellum were negatively correlated with ages. Moreover, we found that the functional connections of hippocampus with its involved network could well predict age. Our findings provide further supporting information that to characterize the functional patterns of hippocampus is crucial to reveal the neural basis of cognitive decline during aging and disease states.

### 4.5 Limitations and prospects

The current research has several limitations. First, how to choose the preference “*P*” value is very hard. Although we set a range of “*P*” values to find the optimal solution in our study, whether a better method to determine the optimal “*P*” should be further investigated. Second, we did not applied this approach to clinical patient data, the stability of our proposed method needs to be further validated since the functional connectivity pattern may changes greatly during disease conditions.

## Supporting information


**Appendix** **S1**: Supporting Information

## Data Availability

All data and code used for data analysis are available upon request.
