# Supplementary material for: Group‐guided individual functional parcellation of the hippocampus and application to normal aging
Source: Hum Brain Mapp. 2021 Sep 16;42(18):5973–84. doi: 10.1002/hbm.25662 (PMC8596973; doi:10.1002/hbm.25662)
Supplement: Supplementary file 1 — Appendix S1: Supporting Information [file HBM-42-5973-s001.docx]

**Supplementary materials**

**Article title:** Group guided individual functional parcellation of the hippocampus and application to normal aging

Jiang Zhang, Dundi Xu, Hongjie Cui, Tianyu Zhao, Congying Chu, Jiaojian Wang

**Figure S1**, Group guided individual parcellation of supplementary motor area (SMA). The group level parcellation of bilateral SMA was first performed and two (K = 2) or three subregions (K = 3) for this area were obtained. The group guided individual parcellation of SMA into two or three subregions was obtained in each subject and the individual parcellation results in five randomly selected subjects were shown.


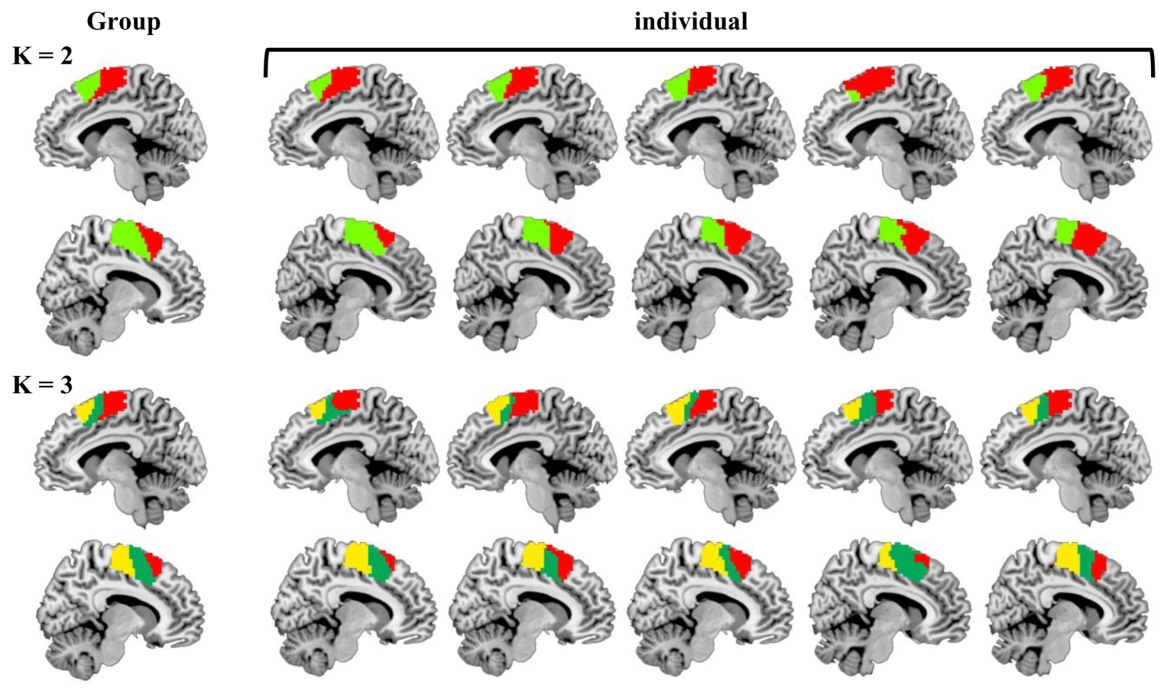


**Figure S2**, Comparison of voxel-level overlap degree. The overlap degree values were obtained by our group guided individual functional parcellation (A) and iteration adjusted individual parcellation (B). C, iteration adjusted individual parcellation results showed higher overlap degree across all the subjects compared with our method.


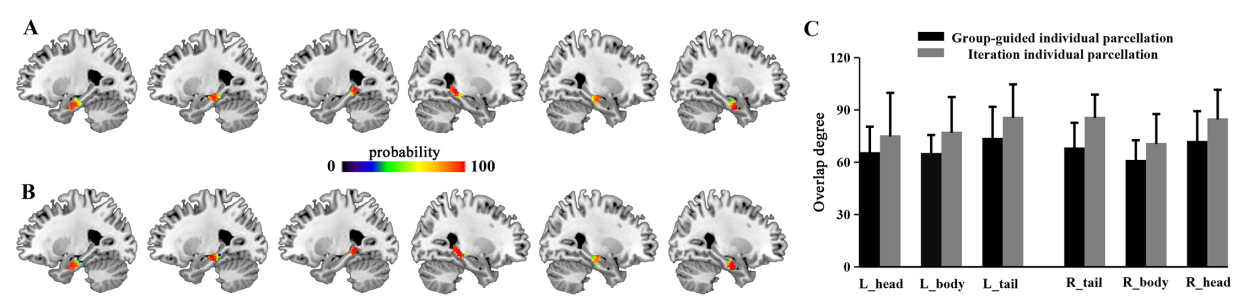


**Figure S3**, Correlations between coactivation patterns, i.e., ALE values, of cognitive memory, episodic recall, and emotion of angry and individual variations of hippocampus parcellation results using iteration adjusted individual parcellation approach. The voxel-level correlation analyses identified significant correlations between individual variations and ALE values of cognitive memory and episodic recall, but no correlation between individual variations and ALE values of emotion of angry was found.


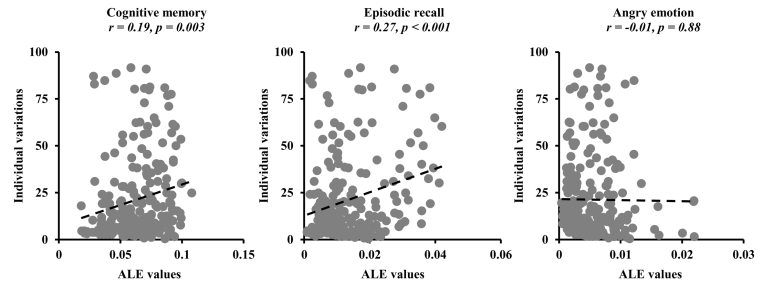


**Figure S4**, Definition of target areas. A, voxel-wise whole brain functional connectivity analysis of hippocampus was performed and one-sample t-test was applied to obtain significantly functionally connected brain areas with hippocampus. B, eight brain areas with significant functional connectivities with hippocampus were defined. The peak MNI coordinates of the eight brain areas were used to draw spheres with 6 mm radium for seed-to-target functional connectivity analysis.


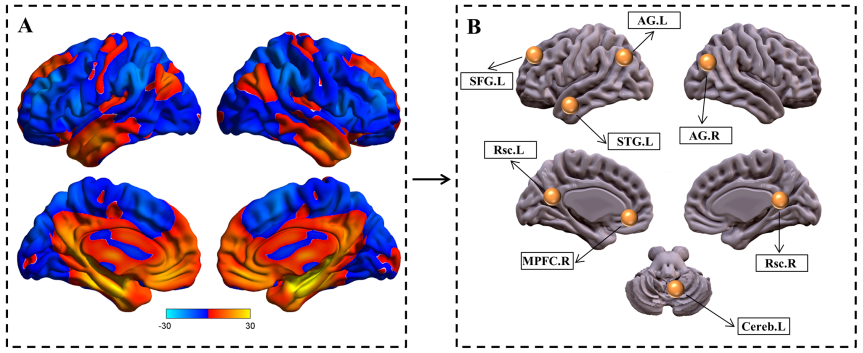


**Figure S5**, Age prediction using individual functional connections calculated by iteration adjusted individual parcellation approach. Although the individual functional connections of hippocampus subregions defined using iteration adjusted individual parcellation approach, the correlation coefficient is lower than that predicted by our method.


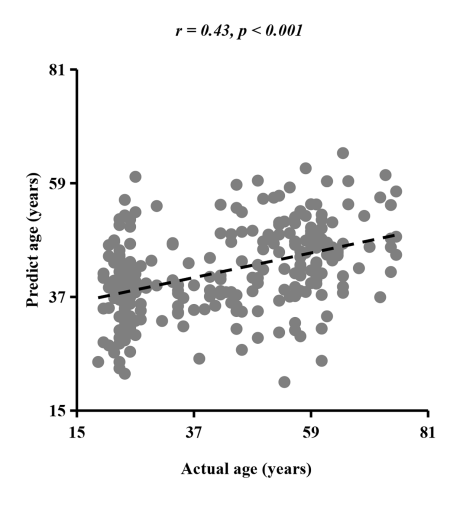


**Figure S6**, Age effects on resting-state functional connectivities. Correlation analyses between individual and group level functional connections between hippocampus subregions and target brain areas were performed. The significant correlations between age and the functional connectivities of hippocampus subregions were found in individual, group level, and both of them after multiple comparisons correction (*p* < 0.05 with Bonferroni corrected).


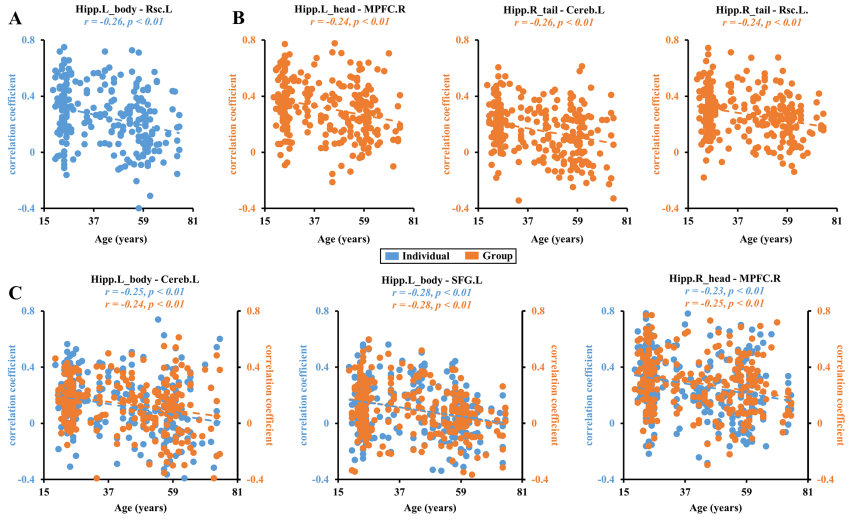


**Table S1:** The peak MNI coordinates of target brain areas defined using whole brain functional connectivity analysis of hippocampus.

| **Brain Regions** | **L/R** | **Abbreviation** | **MNI Coordinates** | | |
| --- | --- | --- | --- | --- | --- |
|  |  |  | **X** | **Y** | **Z** |
| Cerebellum | L | Cereb | -9 | -51 | -48 |
| Superior temporal gyrus | L | STG | -60 | -6 | -21 |
| Medial prefrontal gyrus | R | MPFC | 3 | 42 | -12 |
| Angular gyrus | L | AG | -45 | -72 | 36 |
| Superior frontal gyrus | L | SFG | -18 | 38 | 48 |
| Retrosplenial cortex | L | Rsc | -9 | -52 | 16 |
| Retrosplenial cortex | R | Rsc | 9 | -51 | 9 |
| Angular gyrus | R | AG | 48 | -69 | 36 |

MNI: Montreal neurological institute; L: left hemisphere, R: right hemisphere.
